# Sleep disorders and suicide attempts following discharge from residential treatment

**DOI:** 10.3389/frsle.2023.1173650

**Published:** 2023-09-19

**Authors:** Todd M. Bishop, Westley A. Youngren, John S. Klein, Katrina J. Speed, Wilfred R. Pigeon

**Affiliations:** ^1^VA Center of Excellence for Suicide Prevention, Finger Lakes Healthcare System, Canandaigua, NY, United States; ^2^Department of Psychiatry, University of Rochester Medical Center, Rochester, NY, United States; ^3^Center for Integrated Healthcare, Syracuse VA Medical Center, Syracuse, NY, United States; ^4^Charlie Norwood VA Medical Center, Augusta, GA, United States

**Keywords:** sleep disorder, insomnia, suicide, veteran, sleep-related breathing disorder

## Abstract

**Introduction:**

Suicide is a significant public health concern and its prevention remains a top clinical priority of the Veterans Health Administration. Periods of transition in care (e.g., moving from inpatient to outpatient care) represent a period of increased risk. Sleep disorders are prevalent amongst Veterans and are modifiable risk factor for suicide. The present study examined the relationship of sleep disorders to time to suicide attempt amongst Veterans known to have attempted suicide in the 180 days following discharge from a Mental Health Residential Rehabilitation Treatment Program.

**Method:**

The present sample was comprised of all Veterans enrolled in services with the Veterans Health Administration known to have attempted suicide following discharge from a Mental Health Residential Rehabilitation Treatment Program during Fiscal Years 13 and 14 (*N* = 1,489). To create this sample, electronic medical record data were extracted from two VHA data sources: the Corporate Data Warehouse and the Suicide Prevention Application Network.

**Results:**

Cox regression models revealed that Veterans with a sleep disturbance (*N* = 1,211) had a shorter time to suicide attempt than those without a sleep disturbance [Hazard Ratio (HR) = 1.16, CI (1.02–1.32)]. A subsequent Cox regression model including age, insomnia, nightmare disorder, and alcohol dependence revealed that sleep-related breathing disorders [HR = 1.19, CI (1.01–1.38)], alcohol dependence [HR = 1.16, CI (1.02–1.33)], and age group were associated with increased risk.

**Conclusion:**

Findings indicate that sleep disturbance, primarily driven by sleep-related breathing disorders, was associated with time to suicide attempt in this sample of high-risk Veterans known to have attempted suicide in the 180 days following their discharge from a Mental Health Residential Rehabilitation Treatment Program. These findings reveal an opportunity to reduce risk through the screening and treatment of sleep disorders in high-risk populations.

## Introduction

Suicide remains a leading cause of death within the United States (Drapeau and McIntosh, 2017; Xu et al., 2018) and presents a serious public health concern. Suicide death, however, does not capture the full scope of this pervasive crisis, as for every known suicide, there are an estimated 30 suicide attempts that do not result in death (Han et al., 2016). Thus, the identification of modifiable factors that are associated with suicide remains necessary in order to facilitate the design and implementation of effective initiatives aimed at preventing suicide. When considering situations where a variety of different risk factors may negatively interact, transitions between inpatient and outpatient mental health services may be a target of interest.

### Care transitions

Care transitions, operationally defined as a transition from inpatient to outpatient mental health services, have been identified as a period of elevated risk for suicide (Valenstein et al., 2009; Britton et al., 2015, 2017). The greatest risk is often observed in the month following discharge from the inpatient unit and diminishing over the following 6 months (Chung et al., 2017; Kessler et al., 2020, 2023). Mental Health Residential Rehabilitation Treatment Programs (MHRRTPs) offered by the Veterans Health Administration (VHA) are used to treat Veterans with a wide range of conditions and are a setting where care transitions frequently occur. MHRRTPs target a range of Veteran concerns including homelessness, substance use, and posttraumatic stress disorder (PTSD). In 2012, within the United States, there were an estimated 8,407 beds across 237 MHRRTPs (Northeast Program Evaluation Center, 2012a,b). These programs serve a valuable resource for Veteran care and provide a needed respite for many. Veterans stay on average 50.7 days during a given MHRRTP visit (Northeast Program Evaluation Center, 2012a,b). This time is often focused on stabilization, treatment planning, and skill development centered on addressing the core disorder(s) that served as the basis of the admission and pressing discharge issues such as obtaining housing. Additionally, these care transitions from MHRRTP programs can sometimes be a time of stress and uncertainty, which in turn has the possibility of impacting suicide risk after care transition. Despite this observation, it should also be noted that although suicide is a leading cause of death, it remains a low-base rate behavior and that the vast majority of MHRRTP patients do not go on to attempt suicide in the months following discharge. Nonetheless, examining the health factors of those patients that do attempt suicide, we may reveal risk profiles that suggest the need for special attention. The presence of known risk factors such as sleep disorders is of particular interest.

### Sleep, veterans, and MHRRTPs

Studies of both active duty service members (Mysliwiec et al., 2013) and post-deployment service members reveal an elevated prevalence of sleep disorders compared to the general population (Bramoweth and Germain, 2013). This pattern continues to be observed among Veterans who are impacted by a range of sleep disorders, with insomnia and obstructive sleep apnea being more frequently diagnosed in Veterans than their non-Veteran age-matched counterparts (Bramoweth and Germain, 2013; Ulmer et al., 2015; Alexander et al., 2016).

For one, military service, particularly deployments, have not traditionally been synonymous with developing or maintaining healthy sleep habits (e.g., shift work, increased caffeine intake, trauma exposure). As to Veterans within MHRRTP settings, there are numerous challenges to healthy sleep that are consistent with theoretical models on the development and perpetuation of insomnia disorder. These factors include, but are not limited to, changes in medication regimen, substance detox, changes in one's sleep window due to programming or mandated “lights out” times, disruptive roommates, hospital lighting and temperatures, and room checks by staff throughout the night.

In addition to MHRRTPs not being a particularly conducive environment for sleep, should a Veteran be diagnosed with a sleep disorder, behaviorally based treatment options are not always readily available while enrolled in a MHRRTP. While the VHA has led the nation in the provision of cognitive behavioral therapy for insomnia (CBT-I), the majority of MHRRTPs do not have providers on-site who are trained to provide the therapy, which is problematic given that Veterans receiving MHRRTP care commonly present with insomnia (Bishop et al., 2019b). Unfortunately, there are even fewer providers trained in behavioral health approaches to addressing other sleep concerns such as trauma-related nightmares. Therefore, MHRRTP's can be environments that disrupt healthy sleeping habits while having limited capacities to address sleep-related issues using behavioral interventions. All of this, of course, does not preclude the use of readily available pharmacological interventions for insomnia or indicated interventions for other sleep disorders (e.g., positive airway pressure for obstructive sleep apnea).

### Sleep and suicide

The limited number of behavioral health providers trained in the provision of CBT-I and other sleep-oriented interventions is made even more concerning when one considers the association between sleep disorders and suicidal thoughts and behaviors (Pigeon et al., 2012b, 2016; Bishop et al., 2019a). Previous research has shown sleep disorders are associated with decreased time to death among suicide decedents (Pigeon et al., 2012a), suggesting that the presence of sleep disturbance may serve as an important predecessor to heightened risk of suicide. This is supported by an ample amount of research that demonstrates the strong predictive relationship between sleep disorders and suicide, with disorders such as sleep apnea and insomnia being consistent predictors of suicide attempts.

### Care transitions, sleep, and suicide

As stated, both sleep disorders and care transitions are risk factors for suicide. Considering that sleep disorders are often more prevalent and can be under addressed within programs where care transitions frequently take place (such as MHRRTPs), examining the relationship between care transitions and sleep disorders within programs such as MHRRTPs, may provide novel insights into suicide risk factors. Thus, our study aimed to examine the relationships among sleep disorders and time to suicide attempt following discharge from a MHRRTP setting (care transition) among Veterans with a suicide attempt in the 6-months following discharge.

## Method

### Study sample and data sources

Data for the present analyses were drawn from a parent dataset originally consisting of all Veterans enrolled in VHA services with a documented suicide attempt in FY13–14 with a 1:1 matched case-control group of Veterans with no attempt (*N* = 60,102; Bishop et al., 2019a). Data were extracted from two VHA data sources: the Suicide Prevention Application Network (SPAN) and the Corporate Data Warehouse (CDW). SPAN compiles data on suicide attempts known to VHA (e.g., date and method of attempt; Hoffmire et al., 2016). Since 1999 the CDW has served as the VHA's storehouse of clinical encounter and administrative data. To support the present analyses we extracted treatment utilization, diagnostic (i.e., ICD codes), pharmacy, and demographic data.

The present sample is comprised of Veterans (*N* = 1,489) with a documented suicide attempt in the 180 days post discharge from a MHRRTP during FY13–14 (see Table 1). In line with the current Veteran population, our sample was largely male (92.3%) and identified as White (74.2%) or Black/African American (20.5%). The Veterans in this sample had a mean age of 46.5 years. The most commonly identified eras of service were Vietnam (20.8%), post-Vietnam (29.1%) and the Persian Gulf War or the operations in Iraq and Afghanistan following September 11, 2001 (49.8%).

**Table 1:** Participant characteristics.

	**Documented sleep disturbance (*N =* 1,211)**	**No documented sleep disturbance (*N =* 278)**			
	**N (%)**	***n*** **(%)**	*χ^2^*	***df***	***p***
**Age** ^a^					
18 to 29	166 (13.7)	26 (9.4)	2.77		0.003
30 to 39	217 (17.9)	39 (14.0)			
40 to 49	259 (21.4)	59 (21.2)			
50 to 59	418 (34.5)	114 (41.0)			
60 to 69	146 (12.0)	37 (13.3)			
70 +	5 (0.4)	3 (1.1)			
**Gender**					
Male	1,115 (92.1)	259 (93.2)	0.38	1	0.538
Female	96(7.9)	19 (6.8)			
PTSD	804 (66.4)	125 (45.0)	44.24	1	< 0.001
Depression	1,113 (91.9)	237 (85.3)	11.83	1	< 0.001
Bipolar disorder	384 (31.7)	93 (33.5)	0.32	1	0.574
Schizophrenia	132 (10.9)	50 (18.0)	10.58	1	0.001
Substance use disorder^b^	1,091 (90.1)	251 (90.3)	0.01	1	0.921
Alcohol dependence	986 (81.4)	228 (82.0)	0.05	1	0.818
**Comorbidity score** ^a, c^					
0	715 (59.0)	168 (60.4)	−0.25		0.40
1	280 (23.1)	58 (20.9)			
2	124 (10.2)	31 (11.2)			
3	92 (7.6)	21 (7.6)			
Mental health visits^a, e^	30.5 (43.0) [7]	23.2 (37.2) [0]	−2.91		0.002

^a^Wilcoxon Mann Whitney test *z* statistic reported; ^b^Substance use disorder includes drug dependence and substance abuse apart from alcohol dependence and tobacco use; ^c^Categorized Gagne comorbidity score without weights for psychosis and alcohol; ^d^SRDB, Sleep-Related Breathing Disorder; ^e^Mental health visits within year prior to index visit, mean (standard deviation) [Mdn].

### Measures

#### Days to suicide attempt

Notably, our primary outcome variable is built using suicide attempts observed in the medical record as opposed to ideation, which may have different predictors. The primary outcome for the present study was time from discharge from a MHRRTP to suicide attempt. Suicide attempts were captured via a merger of SPAN and CDW data (i.e., presence of a suicide attempt in either CDW or SPAN was counted as an attempt). Poor overlap has previously been documented between these two monitoring systems (e.g., approximately 41% of suicide attempts appear in both systems) (Hoffmire et al., 2016). Thus, it was decided to extract suicide attempt data from both CDW and SPAN to more fully capture the scope of this outcome. In CDW, suicide attempts were identified by the presence of ICD code E950–E959. In SPAN, events were considered a suicide attempt regardless of whether they resulted in an injury or were interrupted. Any cases of self-injury without suicide intent, as indicated by the event report in SPAN, were excluded.

#### Sleep disorders

ICD-9 codes were extracted from CDW and used identify the presence of nightmare disorder (307.47), sleep-related breathing disorders (327.20, 327.21, 327.23, 327.26, 327.27, 327.29, 780.57) and insomnia (307.41, 307.42, 327.02, V69.4, 291.82, 292.85, 327.01, 780.52, 327.00, 327.8, 307.48, 327.36). We next extracted pharmacy data to identify additional cases where nightmares and insomnia were likely to be present. Participants were considered to be experiencing nightmares if they carried a diagnosis of PTSD and were prescribed Prazosin. In addition, participants were classified as having insomnia if it was documented that they had filled a prescription for a medication at dosages associated with the treatment of insomnia (e.g., non-benzodiazepine “z-drugs,” benzodiazepines, sedative/hypnotics; Lavigne et al., 2019).

#### Multimorbidity/gagne index

Data were extracted on the series of medical conditions (e.g., hypertension, pulmonary disease) used to calculate the Gagne Index (Quan et al., 2005; Gagne et al., 2011), a measure of medical comorbidity. Diagnoses are weighted and used to create a score (ranging from −2 to 26) that has been used in the prediction of mortality risk. Psychosis and alcohol abuse were not included in the algorithm to reduce overlap with covariates. To simplify, scores were then categorized into the following groups: [0 (−2 to 0), 1, 2, 3 (≥3)].

#### Substance use and mental health disorders

ICD codes were used to document the presence of several substance use and mental health disorders including: depression, bipolar disorder, PTSD, schizophrenia, and anxiety disorders. If the medical record of a given participant was found to have both bipolar and depression diagnoses in the assessment period they were classified as having bipolar disorder.

### Statistical analyses

The primary relationship of interest was the comparison of time to suicide attempt following discharge from a MHRRTP among Veterans with and without a sleep disturbance. We also examined the impact of several known drivers of suicide risk including depression, substance use disorder, PTSD, schizophrenia, bipolar disorder, and medical comorbidity. For purpose of analyses, these disorders were considered present if they were documented in the EMR in the 180 days prior to the index date (first suicide attempt in the time period). The 180-day time period was selected to ensure that the diagnoses were current.

We began the modeling process with a univariate Cox model for each predictor of interest. All predictors met the proportional hazards assumption for Cox regression. Predictors that were significantly associated (type 3 test *p* < 0.05) with the hazard rate of attempt in our sample were included in a multivariate model. Backward selection was used to remove predictors that did not contribute to the fit of the multivariate model.

## Results

Of the 1,489 Veterans in our dataset who had attempted suicide following discharge from an MHRRTP program in FY13–14, a total of 1,211 (81.3%) had a documented sleep disorder (or medication for the treatment of a sleep disorder) and 278 (18.7%) did not. In this sample, depression, PTSD, and age were all associated with having a documented sleep disturbance. Chi-square and Wilcoxon Mann Whitney tests did not reveal differences between the sleep disordered and non-sleep disordered groups on biological sex, alcohol dependence, substance use disorder, and medical comorbidity (see Table 1).

In the univariate Cox models, age group, alcohol dependence, substance use disorder, and sleep disturbance were significantly associated with hazard of suicide attempt (see Table 2). These variables were subsequently included in the multivariate model. Backward elimination then determined that substance use disorder did not contribute to the model.

**Table 2:** Hazard ratios by sleep disorder type for veteran suicide attempts following discharge from a MHRRTP in FY13–14 (*N* = 1,488).

	**Univariate analysis**		**Multivariate analysis**	
**Variable**	**Coefficient**	**Hazard ratio (95% CI)**	**Coefficient**	**Hazard ratio (95% CI)**
Insomnia	0.10	1.10 (0.98–1.25)		
SRBD	0.13	1.14 (0.97–1.33)		
Nightmare	0.10	1.10 (0.96–1.26)		
Sleep disturbance	0.15	1.16 (1.02–1.32)	0.14	1.16 (1.02–1.32)
PTSD	0.05	1.05 (0.95–1.17)		
Depression	0.11	1.12 (0.94–1.34)		
Bipolar	0.10	1.10 (0.96–1.23)		
Schizophrenia	0.05	1.05 (0.89–1.23)		
Substance use disorder	0.18	1.20 (1.01–1.42)		
Alcohol dependence	0.13	1.14 (1.00–1.30)	0.15	1.16 (1.02–1.33)
**Comorbidity score** ^a^				
1	−0.07	0.94 (0.83–1.06)		
2	0.00	1.00 (0.84–1.18)		
3	−0.06	0.95 (0.77–1.15)		
**Age group** ^b^				
18–29	−0.037	0.96 (0.51–2.13)	−0.05	0.96 (0.50–2.11)
30–39	−0.288	0.75 (0.40–1.66)	−0.29	0.75 (0.40–1.65)
40–49	−0.330	0.72(0.38–1.59)	−0.35	0.71 (0.38–1.56)
50–59	−0.287	0.75 (0.40–1.65)	−0.31	0.74 (0.39–1.62)
60–69	−0.287	0.73 (0.38–1.61)	−0.34	0.71 (0.37–1.57)

CI, Confidence interval; SRBD, sleep-related breathing disorder; ^a^Reference group is comorbidity score 0; ^b^Reference group is age group 70+.

The final model included age group, sleep disturbance, and alcohol dependence. According to Hazard ratio estimates patients with a sleep disturbance (diagnosis or prescription for insomnia, SRBD, or nightmare disorder) had a 16% higher hazard of a suicide attempt at each observed time point throughout the 180 days following domiciliary discharge than those without a sleep disturbance. Having alcohol dependence also increased the hazard of suicide attempt by 16% during the year after discharge relative to those without alcohol dependence.

While none of the components of the specific sleep disorders met inclusion criteria (*p* < 0.05) for the multivariate model separately, a second multivariate model examined how each disorder was driving the effect of sleep disturbance. This model included age group and alcohol dependence. Backward selection removed insomnia and nightmares from this model. SRBD, age group, and alcohol dependence remained, suggesting that SRBD was the primary driver of the sleep disturbance effect.

As both ICD codes and pharmacological interventions (Rx) were used in the operationalization of the sleep variables, a series of sensitivity analyses were conducted to determine whether any component (ICD codes or pharmacological intervention) disproportionately impacted the results. There were no changes to the significance of the reported relationships and parameter estimates remained similar regardless of which operationalization of sleep problems were employed [Insomnia (ICD or Rx = 0.098); (ICD only = 0.089); (Rx only = 0.096)].

## Discussion

Relationships among insomnia, nightmares, sleep-related breathing disorders and suicide risk were examined among Veterans with a known suicide attempt following discharge from a MHRRTP residential program during FY13–14. In this population it was found that those with a documented sleep disorder (including those undergoing pharmacological intervention for disordered sleep) had a greater hazard of attempting suicide sooner following discharge than their non-sleep disordered counterparts. These findings held even when accounting for factors known to be associated with increased suicide including age group, alcohol dependence, SUD, schizophrenia, bipolar disorder, depression, and PTSD. This aligns with previous findings among a sample of Veteran suicide decedents, for whom time to suicide was shorter among those with a sleep disturbance (Pigeon et al., 2012a).

Relationships among sleep disorders and suicide risk are impacted by numerous factors. It is not simply the presence or absence of a given disorder that is associated with increased suicide risk, but rather the deleterious impact of disrupted sleep on multiple aspects of psychological and biological functioning. While not every individual who has trouble sleeping will contemplate suicide, the impact of disrupted sleep is likely to be more pronounced during periods of acute adjustment (e.g., care transitions, inpatient hospitalizations). Importantly, improvements in sleep health, and any subsequent benefits to the reduction of suicide risk, can also be impacted by the stability of the post-discharge setting and treatment regimen. Upon discharge from residential services patients may find themselves in housing and sleeping situations that have negative impacts on their ability to enact positive changes in their sleep that were adopted during more intensive treatment. Clinicians focused on sleep must often spend additional effort to plan for post residential care, set expectations, and adjust the treatment plan for it to be successful. Relatedly, the functional impact of untreated sleep disorders may inhibit one's ability to enact other behavioral health interventions adopted during residential care. For example, decreased executive functioning as a result of poor sleep could restrict one's ability to enact cognitive behavioral strategies to address drivers of suicide risk associated with other disorders such as depression or PTSD. Hence the importance of identifying and treating sleep disorders throughout the continuum of care.

### Clinical implications

Care transitions may represent a period of increased risk for suicide. These findings reveal an opportunity to reduce risk through addressing commonly occurring sleep disorders. Not only do sleep disorders represent a modifiable risk factor for suicide, but there are also highly effective treatments that target them, such as positive airway pressure for sleep apnea. The high prevalence of sleep disorders alone in this population speaks to the need to focus on the assessment and treatment of sleep among MHRRTP patients. The urgency of such assessment and treatment (whatever the treatment modality) is enhanced given the data that suggests that presence of a sleep disorder reduces time to event among those that attempt suicide in the post discharge period. Sleep should not only be addressed in treatment and post-discharge planning, but also in the development of safety planning (Hochard et al., 2015).

Targeting sleep may also serve an important role in fostering resiliency during care transitions and fostering long-term recovery. Sleep disorders represent a low stigma treatment target that can often be addressed with brief interventions that may lend themselves to delivery in multiple settings. MHRRTPs should devote sufficient time and resources for training in behavioral health interventions targeting sleep in order to complement readily available pharmacological interventions. For example, brief cognitive behavioral therapy for insomnia (bCBTi), brief behavioral therapy for insomnia (BBTI), image rehearsal therapy (IRT), and the introduction of treatments for sleep apnea (e.g., positive airway pressure), are all things that can be implemented in short periods of time, relative to other clinical interventions, and in residential settings.

### Limitations and future directions

There are some limitations of note to the present analysis. First, as our sample is composed MHRRTP patients who were known to have attempted suicide during our study time period, the sample is relatively severe. While it is of great importance to understand the trajectory to suicide attempt, these analyses may not be representative of the larger MHRRTP patient pool. Second, insomnia disorder has historically under documented in the medical record. Thus, while we attempt to capture additional cases of insomnia with our medication algorithm, the prevalence of this disorder is likely underrepresented in our sample. Although this increases the likelihood that we observe more of the existing cases of sleep disorders, it also means that some participants in the sample will be receiving treatment (e.g., pharmacological therapy for insomnia) when others are not. As mentioned above at the end of the Results section, sensitivity analyses were conducted to account for this, but in future studies a more detailed reporting of the association between time of diagnosis and treatment initiation for sleep disorders would be beneficial. Third, in the current study we use a dichotomous variable to indicate whether a participant had a given condition (e.g., insomnia) in the 180 days preceding discharge from the residential unit. We do not provide a more nuanced time of diagnosis in relationship to discharge, the timing of which could have implications for the findings. Fourth, it is important to note that this study has examined suicide attempts as its primary outcome, as opposed to death. Examinations of suicide-related behavior (as opposed to ideation) are an advance to the literature; however, it may not be automatically assumed that the risk factors for suicide attempt, and their associated effect sizes, have an equivalent impact when examining suicide as an outcome. Fifth, the use of Cox Regression and hazard ratios provides us with a unitless measure of the effect that a given predictor has on the outcome. However, hazard ratios do not generally lend themselves to the traditional interpretation of small, medium, or large effects sizes as can be done with other analytical methods. Finally, data were drawn from FY13–14 and may not reflect current realities including enhanced suicide prevention practices adopted across VHA including in MHRRTP settings.

We envision several, potentially key, future directions for this work. First, the prospective examination of these relationships, starting with sleep and moving forward in order to obtain estimates related to the broader sleep-disordered population. Second, incorporation of sleep architecture data into analyses to deepen our understanding of the potential physiological underpinnings of the sleep-suicide relationship. Third, the addition of mortality data. Fourth, identification and testing of potential interventions during care transitions to reduce suicide risk. The latter of these may be particularly interesting to investigate in a replication of the current study with data from current fiscal years as the VHA continues to implement enterprise-wide changes to improve transitions in care (Department of Veterans Affairs., 2008; Britton et al., 2017).

## Conclusion

Overall, our results indicate that Veterans with a documented sleep disorder had a greater likelihood of attempting suicide sooner following discharge than their non-sleep disordered counterparts. These results are meaningful, as they identify conditions that when sufficiently treated, could directly reduce the likelihood of a Veteran attempting or dying by suicide. While the findings are not fully generalizable to the broader Veteran or civilian population, they provide clinically useful information and further document the close relationship between sleep and suicide.

## Data availability statement

The data analyzed in this study is subject to the following licenses/restrictions: the dataset is derived from the Department of Veterans Affairs electronic medical record and is not available for public use. Requests to access these datasets should be directed to todd.bishop@va.gov.

## Ethics statement

The studies involving humans were approved by Syracuse VA Institutional Review Board. The studies were conducted in accordance with the local legislation and institutional requirements. Written informed consent for participation was not required from the participants or the participants' legal guardians/next of kin in accordance with the national legislation and institutional requirements.

## Author contributions

TB, WY, KS, and WP were each involved in the conceptualization of the study and analysis. JK performed the statistical analysis and drafted the results section. All authors contributed substantively to the creation of this manuscript, writing, and editing of the manuscript.
